# AET-FRAP—A Periodic Reshape Transformer Framework for Rock Fracture Early Warning Using Acoustic Emission Multi-Parameter Time Series

**DOI:** 10.3390/s25247580

**Published:** 2025-12-13

**Authors:** Donghui Yang, Zechao Zhang, Zichu Yang, Yongqi Li, Linhuan Jin

**Affiliations:** 1 School of Coal Engineering, Shanxi Datong University, Datong 037003, China; ydhname@163.com (D.Y.);; 2 The Cultivation Base of Shanxi Key Laboratory of Coal Mine Water Jet Technology and Equipment, Shanxi Datong University, Datong 037003, China

**Keywords:** rock fracture, deep learning, time series prediction, acoustic emission, early warning

## Abstract

**Highlights:**

**What are the main findings?**

- A novel AET-FRAP framework transforms 1D AE signals into 2D tensors for precise energy prediction.
- Collaborative Cosine Similarity and Kurtosis metrics define quantitative fracture warning triggers.

**What are the implications of the main findings?**

- The framework achieves superior accuracy (R^2^ ≈ 1), outperforming LSTM in rock fracture forecasting.
- It provides a reliable, noise-resistant early warning solution for deep underground engineering safety.

**Abstract:**

The timely identification of rock fractures is crucial in deep subterranean engineering. However, it remains necessary to identify reliable warning indicators and establish effective warning levels. This study introduces the Acoustic Emission Transformer for FRActure Prediction (AET-FRAP) multi-input time series forecasting framework, which employs acoustic emission feature parameters. First, Empirical Mode Decomposition (EMD) combined with Fast Fourier Transform (FFT) is employed to identify and filter periodicities among diverse indicators and select input channels with enhanced informative value, with the aim of predicting cumulative energy. Thereafter, the one-dimensional sequence is transformed into a two-dimensional tensor based on its predominant period via spectral analysis. This is coupled with InceptionNeXt—an efficient multiscale convolution and amplitude spectrum-weighted aggregate—to enhance pattern identification across various timeframes. A secondary criterion is created based on the prediction sequence, employing cosine similarity and kurtosis to collaboratively identify abrupt changes. This transforms single-point threshold detection into robust sequence behavior pattern identification, indicating clearly quantifiable trigger criteria. AET-FRAP exhibits improvements in accuracy relative to long short-term memory (LSTM) on uniaxial compression test data, with R^2^ approaching 1 and reductions in Mean Squared Error (MSE), Root Mean Squared Error (RMSE), and Mean Absolute Error (MAE). It accurately delineates energy accumulation spikes in the pre-fracture period and provides advanced warning. The collaborative thresholds effectively reduce noise-induced false alarms, demonstrating significant stability and engineering significance.

## 1. Introduction

Pillar mining in subterranean engineering reinforces the ceiling and floor of mine workings by creating pillars, facilitating ore extraction [1]. Reasonable pillar layout and stability control are critical for coordinated mining and roadway safety [2]. These pillars must maintain stability during the mining operation, supporting the stresses and deformations of the overlying rock mass. Mining disturbances consistently modify the stress state of the adjacent rock. Elevated stresses may induce tensile stripping or shear failure at the pillar’s free surface or the discontinuous fracture surface, consequently jeopardizing industrial safety in mining engineering [3]. Most underground engineering disasters are caused by brittle failure of rock masses. Rock instability poses significant geological hazard risks in underground engineering [4]. Monitoring and identifying pillar failure is crucial for early warning and disaster prevention [5], yet understanding the complex mechanisms of rock instability events remains a major challenge in rock mechanics. Recent studies have utilized catastrophe theory, such as the folding catastrophe model, to elucidate the nonlinear dynamic evolution and disaster-causing mechanisms of spalling rock bursts [6]. Consequently, researchers have conducted extensive studies to develop effective prediction and early-warning systems [7,8].

Approaches to rock fracture prediction can be broadly classified into two types of methodologies [9]: long-term prediction and short-term prediction. Long-term prediction is typically employed during the engineering design phase to assess the likelihood and severity of rock fracture events [10]. This entails assessing fracture likelihood based on the stress environment, integrating elements such as the strain energy storage index [11], the rock brittleness index [12], the tangential stress concentration factor [13], failure duration [14], and other prevalent metrics. Empirically, one or more of these indices are frequently utilized for determination [15]. In contrast, short-term prediction is utilized throughout the building phase of the project, employing field monitoring data to anticipate likely fracture sites and their severity. Through the analysis of dynamic alterations in monitoring data, precursor attributes of fracture can be discerned, including acoustic emission (AE) [16], microseismic activity [17], electromagnetic radiation [18], and infrared (IR) signals [19].

The strain energy released during rock fracture is emitted as sonic waves, which can be captured using acoustic emission (AE) techniques [20]. These waves can delineate the characteristics of microcrack formation, encompassing quantity, intensity, and propagation direction. Numerous AE techniques have been established to clarify failure mechanisms [21], including the seismic b-value method [22], source mechanism inversion [23], and microcrack categorization based on RA (rising time/amplitude) and AF (average frequency, count/duration). The RA-AF approach creates a framework for qualitatively differentiating tensile microcracks from shear microcracks by statistically analyzing the RA and AF values of acoustic emission signals [24,25]. Zhang and Deng [26] confirmed the precision of their method by examining the predominant frequencies of acoustic emission waveforms during uniaxial compression testing. This methodology has been utilized to identify failure mechanisms in indirect tensile, three-point bending, modified shear, and uniaxial compression tests [27]. The approach is sensitive to the selection of the signal window, and its precursor features frequently depend on operating conditions [28,29]. Its application necessitates reevaluation as geological conditions alter. Therefore, implementing multi-indicator cross-validation and developing a comprehensive early-warning system are essential for improving early-warning effectiveness.

In recent years, the utilization of deep learning for short-term prediction and early warning has become a significant study area. In contrast to conventional artificial neural network (ANN) models [30], backpropagation (BP) neural network models exhibit accelerated convergence rates and enhance prediction accuracy to over 90% [31]. Extreme learning machine (ELM) models, optimized by genetic algorithms (GAs), have diminished prediction errors to under 5% [32]. The C5.0 decision tree classifier attained a prediction accuracy of 95.98% for rock burst occurrence and 91.10% for categorizing rock burst intensity levels [33]. Moreover, long short-term memory (LSTM) and recurrent neural networks (RNNs) utilized in the time series analysis of coal mine electromagnetic radiation signal amplitudes successfully discerned the precursor traits of rock bursts [34]. Nevertheless, the majority of short-term prediction studies predominantly depend on the monitoring of in situ microseismic data [35], employing microseismic characteristics to discern, categorize, and forecast the intensity of rock fractures. The precise forecasting of rock fractures continues to pose a considerable challenge in rock engineering.

In conclusion, existing rock fracture early-warning systems still face two major challenges. Firstly, in terms of predictive modeling, traditional deep learning methods (such as LSTM) often struggle to capture the complex, highly non-stationary characteristics of acoustic emission signals. This leads to prediction delays or insufficient accuracy during critical rapid fracture stages. Secondly, concerning early-warning strategies, systems frequently lack clear, quantifiable methodologies for selecting warning indicators, identifying precursors, and setting warning thresholds [36]. They typically rely on expert judgement and static single-point thresholds [37], rendering them highly susceptible to noise interference and false alarms.

This study introduces an advanced early-warning framework for rock fracture prediction, termed AET-FRAP (Acoustic Emission Transformer for FRActure Prediction), which encompasses a systematic approach to indicator selection, temporal forecasting, and threshold determination. This approach initially identifies the most informative combinations of features based on the variability and sudden change characteristics of several acoustic emission parameters during the pre-fracture phase. It subsequently utilizes Empirical Mode Decomposition (EMD) and Fast Fourier Transform (FFT) to perform a comprehensive periodic analysis of these feature sequences, uncovering their inherent temporal patterns. The primary forecast target is cumulative energy, guaranteeing the efficacy of early-warning indicators from the beginning. A high-performance AET-FRAP time series prediction model was developed based on this basis. The model’s primary innovation is the implementation of a ‘periodic reshaping’ method, which converts one-dimensional acoustic emission signals into two-dimensional tensors to capture intra-period and inter-period fluctuations across various scales, thus facilitating enhanced feature learning. It utilizes lightweight InceptionNeXt modules for efficient processing, finally attaining high-precision predictions of strongly non-stationary signals, with performance markedly exceeding that of standard models. Moreover, to improve the dependability of early-warning systems, this framework discards traditional single-point thresholding. It analyzes the dynamic properties of high-precision prediction sequences, employing cosine similarity and kurtosis to jointly identify sudden changes in behavioral patterns. This paradigm significantly improves precursor detection by defining a clear, quantified collaborative early-warning threshold, hence reducing noise-induced false alarms. It consequently offers an effective and pragmatic alternative for early warning of rock fractures.

## 2. Methodology

### 2.1. Early-Warning Framework for Rock Fracture

**Figure 1:**
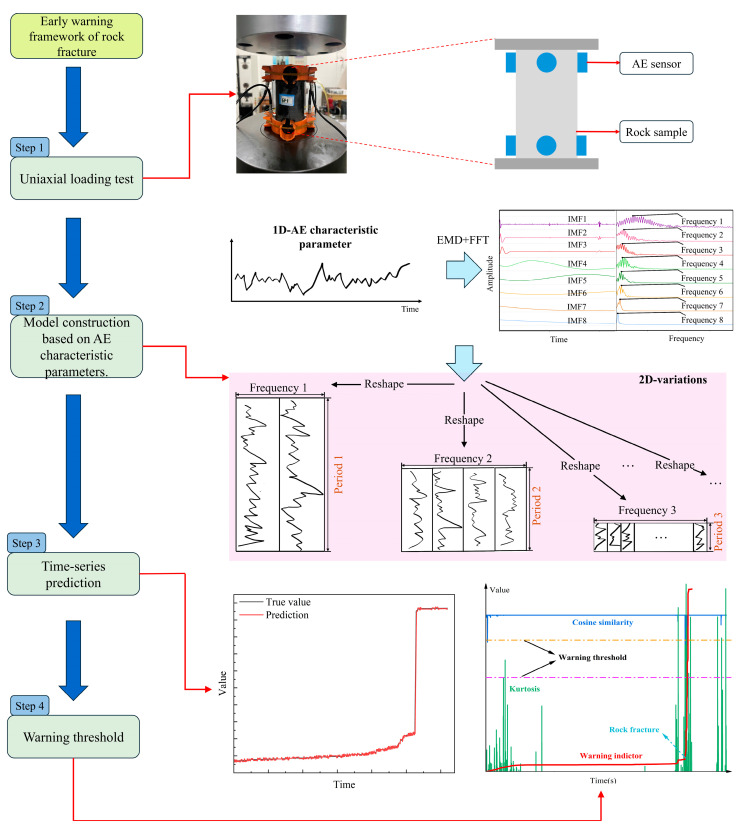
Intelligent prediction framework flowchart for rock fracture.

Figure 1 shows that the proposed rock fracture early-warning framework consists of four processes. Uniaxial loading tests serve to simulate the rock-failure process (Step 1). The loading system and acoustic emission (AE) monitoring system function in synchrony during the test to record a comprehensive time series of acoustic emission parameters, such as ring count, amplitude, and rise time. To address the non-stationary characteristics of the AE signal, a combined analysis method utilizing EMD (Empirical Mode Decomposition) and FFT (Fast Fourier Transform) was employed for periodicity analysis to identify appropriate early-warning indicators (Step 2). The chosen AE early-warning indicators were subsequently incorporated into a time series prediction model to forecast trends in future time intervals (Step 3). The residual similarity was utilized to establish the warning threshold for the indicators (Step 4). This integrated early-warning framework serves as a stable and reliable system for monitoring the stability of engineered rock masses.

This study presents an early-warning method that identifies suitable acoustic emission feature parameters for the prompt detection of rock fracture. Utilizing deep learning’s time series modeling capabilities, the model identifies the progression of anomalies at an earlier stage, thereby significantly increasing the warning lead time and effectively reducing the losses associated with rock fractures. The quantitative establishment and implementation of warning thresholds concurrently enhance the universality and reliability of this early-warning system.

### 2.2. Periodic Analysis of Acoustic Emission Characteristic Signals

**Figure 2:**
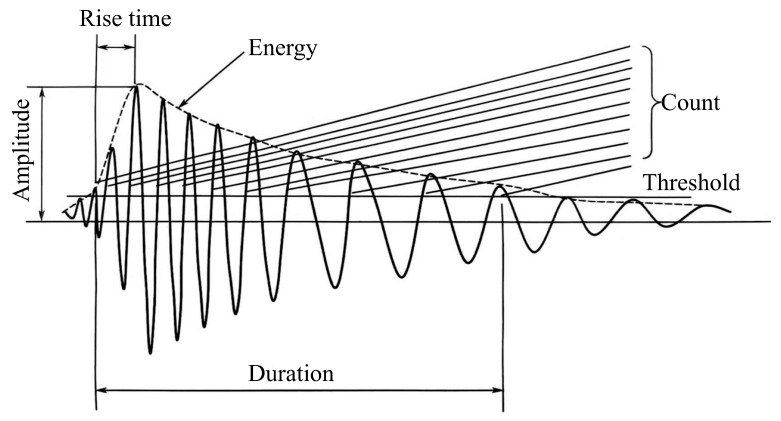
AE characteristic parameters.

The acoustic emission signals obtained during uniaxial loading tests, along with synchronously calculated temporal data for characteristics such as ringing counts, amplitude, and rise time, are illustrated in Figure 2. The acoustic emission characteristic data were preprocessed. Due to the nonuniform sampling of the signals collected during testing, cubic spline interpolation was utilized to convert the raw data into a uniformly sampled format. The resampling frequency was established according to the effective bandwidth of the signal and the Nyquist sampling theorem, ensuring the retention of essential signal information while minimizing unnecessary redundant sampling. The EMD method was subsequently applied to decompose the uniformly sampled acoustic emission feature signals. Empirical Mode Decomposition (EMD) was used to adaptively decompose complex non-stationary signals into multiple intrinsic mode functions (IMFs), with each function representing local signal characteristics across varying temporal scales [38]. The progressive extraction of the IMF components enabled us to effectively separate the multiscale periodic components within the signal. After the acquisition of each IMF component, periodic analyses were conducted on each component individually.

(1) $$\mathrm{IMF}=\left\{c_{1},c_{2},\cdots ,c_{3}\right\}=\mathrm{EMD}\left(\mathrm{Avg}\left(X_{1D}\right)\right)$$

(2) $$F_{\mathrm{sum}}=\sum_{i=1}^{n}\left|\mathrm{FFT}\left(c_{i}\right)\right|$$

(3) $$\left\{f_{1},\cdots ,f_{k}\right\}=\mathrm{argTopk}\underset{f\in \left\{1,\cdots ,\left[\frac{T}{2}\right]\right\}}{\left(F_{\mathrm{sum}}\right)}$$

(4) $$p_{i}=\left[\frac{T}{f_{i}}\right],\;i\in \left\{1,\;\cdots ,k\right\}$$

For a time series acoustic emission signal of the length, *T*, with *C* features, the original 1D structure is represented as $X_{1D}\in R^{T\times C}$, where $\mathrm{Avg}\left(\cdot \right)$ indicates averaging across both the batch and the feature dimensions. $\mathrm{FFT}\left(\cdot \right)$ and $\mathrm{EMD}\left(\cdot \right)$ denote Fast Fourier Transform and Empirical Mode Decomposition, respectively. The cumulative spectral analysis method, as outlined in Equation (2), utilizes different IMF components to represent oscillatory characteristics across various temporal scales within the signal. The individual IMF components derived from Empirical Mode Decomposition were subjected to a Fast Fourier Transform. The amplitude spectra were accumulated to create a composite amplitude spectral representation, which was used to enhance the true periodic signal in the frequency domain. Given the sparsity of frequencies, to prevent the presence of noise from meaningless high-frequency components [39,40], we selected only the top-*k* amplitude values to identify the most significant frequency, $\left\{f_{1},\cdots ,f_{k}\right\}$, thereby minimizing noise from irrelevant high-frequency components, where *k* is a tunable hyperparameter. We computed the frequencies solely within $\left\{1,\;\cdots ,\frac{T}{2}\right\}$ due to the conjugate nature of the frequency domain. We calculated the corresponding *k*-period lengths, $\left\{p_{1},\cdots ,p_{k}\right\}$, from the selected significant frequencies. Equations (1)–(4) are summarized as follows:

(5) $$F_{\mathrm{sum}},\left\{f_{1},\cdots f_{k}\right\},\left\{p_{1},\cdots p_{k}\right\}=\mathrm{Period}\left(X_{1D}\right)$$

### 2.3. Analysis of Resampling Methods

As the original acoustic emission (AE) parameters were recorded as discrete events with nonuniform time intervals, resampling was used as a prerequisite for applying the EMD algorithm and the InceptionNeXt model, both of which require uniformly sampled time series inputs. This study employs cubic spline interpolation to reconstruct continuous signal waveforms. To verify that the observed periodicity constitutes an intrinsic physical property of the rock-fracture process, rather than being an artifact introduced by specific interpolation techniques, we conducted a comparative analysis with linear interpolation. The hypothesis posited that, if periodicity represents a genuine characteristic of the data, then the peak principal frequency should remain unchanged, regardless of the resampling method employed. We reprocessed the AE sequences using linear interpolation and performed an identical EMD-FFT analysis workflow.

As shown in Table 1, the primary periodicity groups identified using both methods are identical. For the representative sample, 3F1, cubic spline interpolation and linear interpolation yielded precisely the same primary periodicity. This consistency confirms that the detected multiscale periodicity constitutes a physical characteristic of the damage evolution process, unaffected by high-frequency discrepancies between interpolation schemes. Furthermore, comparative experiments demonstrate that interpolation methods influence downstream model training. The InceptionNeXt model trained on cubic spline data outperformed the linear interpolation baseline, achieving an R^2^ of 0.9988 and an MSE of 1.2 × 10^−4^. In contrast, the model using linearly interpolated inputs yielded a slightly lower R^2^ of 0.9852 and a higher MSE of 3.5 × 10^−4^. This improvement can be attributed to the second-order derivative continuity of cubic splines, which provides smoother gradients to convolutional layers. This facilitates model convergence more effectively than the non-differentiable spikes introduced by linear interpolation.

Based on these findings, we selected cubic spline interpolation as the optimal preprocessing method to simultaneously ensure the fidelity of periodic analysis and the accuracy of predictions.

### 2.4. Time Series Prediction Model

**Figure 3:**
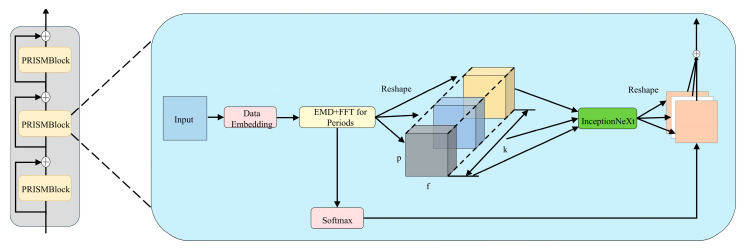
Overall architecture of AET-FRAP.

In this study, we present a temporal model specifically designed for acoustic emission characteristic signals, integrating focused improvements derived from the TimesNet methodology for the analysis of one-dimensional time series data [41]. The model’s fundamental architecture consists of multistage PRISM-Block (Periodic Reshape Inception for Spectral Modelling) modules arranged in a residual configuration [42], as depicted in Figure 3. For $X_{1D}\in R^{T\times C}$, a one-dimensional time series of length *T*, the original features are embedded into a higher-dimensional space, $X_{1D}^{l-1}\in R^{T\times d_{\mathrm{model}}}=\mathrm{Embed}\left(X_{1D}\right)$, through an embedding layer. In the *l*-th layer of AET-FRAP, the input is $X_{1D}^{l-1}\in R^{T\times d_{\mathrm{model}}}$, with $d_{\mathrm{model}}$ representing the feature vector dimension following each time step’s passage through the embedding layer. This embedding layer serves as a robust module that maps raw time series data into a high-dimensional space, maintaining the temporal positional information of the sequence. This yields comprehensive feature representations for the following PRISM-Block.

The period length of the deep feature $X_{1D}^{l-1}$ is calculable using Equation (5). Using the calculated period length, the 1D time series can be transformed into multiple 2D tensors, as illustrated in Equation (6), where ${\mathrm{Reshape}}_{p_{i},f_{i}}\left(\cdot \right)$ denotes the reorganization of the 1D sequence into a 2D format. $p_{i}$ and $f_{i}$ represent the number of rows and columns, respectively, in the transformed two-dimensional tensor, while $\mathrm{Padding}\left(\cdot \right)$ indicates the padding of the time series along the time dimension with zeros to ensure divisibility by $p_{i}$. $X_{2D}^{i}$ denotes the *i*-th time series, reconstructed based on frequency, $f_{i}$, with the rows representing changes within the corresponding cycle length, $p_{i}$, and columns indicating cross-cycle variations (specifically, changes occurring simultaneously across different cycles). Ultimately, k two-dimensional tensors, $\left\{X_{2D}^{1},\cdots ,X_{2D}^{k}\right\}$, are derived from the chosen frequency and its associated cycle. This type of two-dimensional tensor can be efficiently processed using two-dimensional convolution kernels.

(6) $$X_{2D}^{i}={\mathrm{Reshape}}_{p_{i},f_{i}}\left(\mathrm{Padding}\left(X_{1D}\right)\right),\;i\in \left\{1,\;\cdots ,k\right\}$$

(7) $${\hat{X}}_{2D}^{l,i}=\mathrm{InceptionNeXt}\left(X_{2D}^{l,i}\right),\;i\in \left\{1,\;\cdots ,k\right\}$$

**Figure 4:**
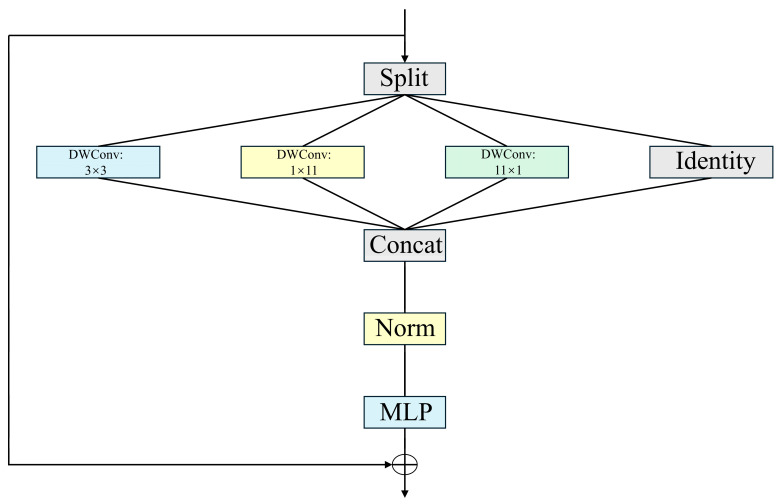
InceptionNeXt block.

Here, to prevent potential frequency-aliasing collisions in the multiperiod analysis, the AET-FRAP framework employs a parallel processing strategy. Rather than blending different frequency components, we construct *k*-independent 2D tensor channels, with each being strictly anchored to a primary period, as determined by EMD-FFT. These channels are subsequently fed into separate InceptionNeXt modules for parallel feature extraction. This design physically isolates distinct frequency components, fundamentally preventing feature-aliasing processes within the deep network. After reshaping, the 2D tensor is input into an Inception block [43]. To optimize the computational resources utilized by EMD, we implement a novel Inception block with the aim of decreasing computational complexity, as defined in $\mathrm{InceptionNeXt}\left(\cdot \right)$ [44] and illustrated in Equation (7). This reduces computational complexity via kernel decomposition and depthwise separable convolutions. The Inception architecture effectively captures multiscale features via parallel convolutions across multiple branches. Larger convolutional kernels lead to a notable decrease in processing speed. The results from InceptionNeXt suggest that certain channels should be maintained in their original state while applying depthwise convolutions exclusively to a selected subset of channels, as demonstrated in Figure 4. The input $X\in R^{B\times C\times H\times W}$ is categorized into four groups based on the channel dimension.

(8) $$X_{\mathrm{hw}},X_{w},X_{h},X_{\mathrm{id}}=\mathrm{Split}\left(X\right)=X_{:,:g},X_{:g:2g},X_{:2g:3g},X_{:3g:}$$

The variable g represents the number of channels in the convolutional branch, while the number of split channels, $g=\tau_{g}C$, is established by defining the ratio, $\tau_{g}$. The divided input is processed through various parallel branches:

(9) $$\begin{array}{c} X_{\mathrm{hw}}^{\prime }={\mathrm{DWConv}}_{k_{s}\times k_{s}}\left(X_{\mathrm{hw}}\right),\\ X_{w}^{\prime }={\mathrm{DWConv}}_{1\times k_{b}}\left(X_{w}\right),\\ X_{h}^{\prime }={\mathrm{DWConv}}_{k_{b}\times 1}\left(X_{h}\right),\\ X_{\mathrm{id}}^{\prime }=X_{\mathrm{id}} \end{array}$$

In Equation (9), $k_{s}$ represents a small square convolution kernel with a default size of 3, $k_{b}$ indicates a strip-shaped convolution kernel with a default size of 11, and $\mathrm{DWConv}\left(\cdot \right)$ denotes depth convolution. The outputs from each branch are then subsequently cascaded.

(10) $$X^{\prime }=\mathrm{Concat}(X_{\mathrm{hw}}^{\prime },X_{w}^{\prime },X_{h}^{\prime },X_{\mathrm{id}}^{\prime })$$

Table 2 presents the computational complexity associated with standard convolutions, deep convolutions, and Inception deep convolutions. The parameters and FLOPs of traditional and deep convolutions demonstrate a quadratic relationship with kernel size *k*, while Inception deep convolutions exhibit a linear relationship with *k*.

The subsequent step entails transforming the 2D tensor, ${\hat{X}}_{2D}^{l,i}$, into a 1D representation, ${\hat{X}}_{1D}^{l,i}\in R^{T\times d_{\mathrm{model}}}$. Due to the padding of zeros at the sequence end during the 2D conversion, it is necessary to utilize $p_{i}\times f_{i}$ to resize the sequence, of length D, back to its original length, T.

(11) $${\hat{X}}_{1D}^{l,i}=\mathrm{Trunc}\left({\mathrm{Reshape}}_{1,\left(p_{i}\times f_{i}\right)}\left({\hat{X}}_{2D}^{l,i}\right)\right),\;i\in \left\{1,\;\cdots ,k\right\}$$

To fuse k-distinct 1D representations, $\left\{{\hat{X}}_{1D}^{l,1},\cdots ,{\hat{X}}_{1D}^{l,k}\right\}$, at the subsequent layer, we consider that the amplitude indicates the relative importance of the chosen frequencies and periods [45], which corresponds to the significance of each remodeled 2D tensor. Thus, we aggregate the composite amplitude spectrum, as follows:

(12) $${\hat{F}}_{{\mathrm{sum},f}_{1}}^{l-1},\cdots ,{\hat{F}}_{{\mathrm{sum},f}_{k}}^{l-1}=\mathrm{Softmax}\left({\hat{F}}_{{\mathrm{sum},f}_{1}}^{l-1},\cdots ,{\hat{F}}_{{\mathrm{sum},f}_{k}}^{l-1}\right)$$

(13) $$X_{1D}^{i}=\sum_{i=1}^{k}{\hat{F}}_{{\mathrm{sum},f}_{1}}^{l-1}\times {\hat{X}}_{1D}^{l,i}$$

**Figure 5:**
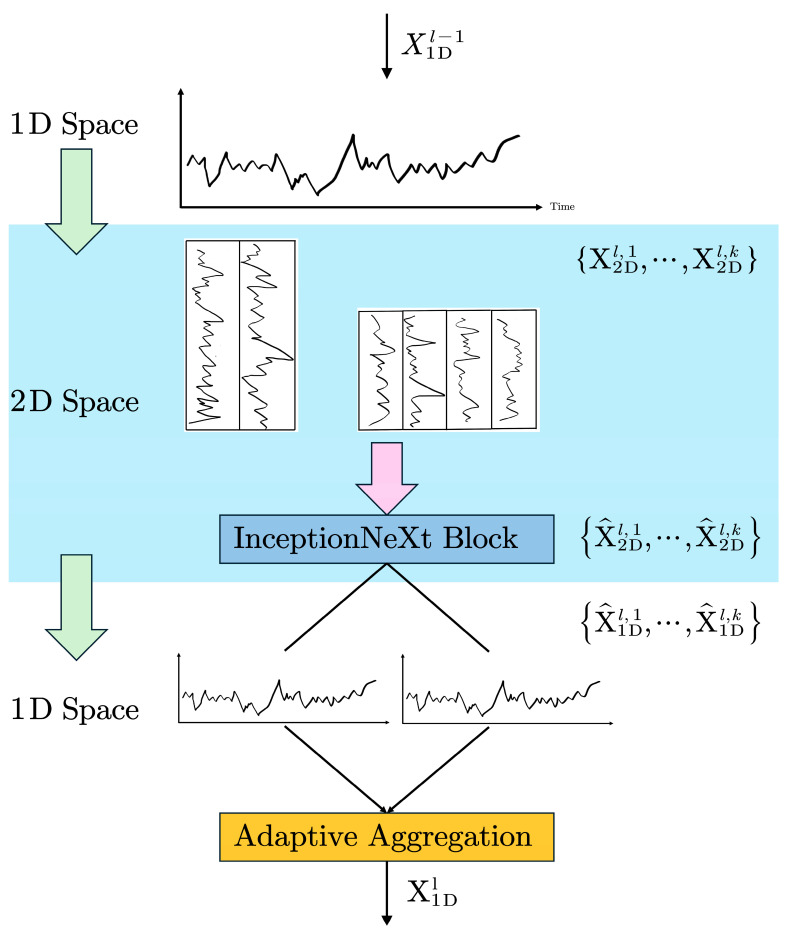
PRISM-Block: 1D–2D reshaping process.

PRISM-Block effectively captures multiscale temporal variations at the two-dimensional level in parallel, as intra-period variation and inter-period variation are already embedded within multiple structured two-dimensional tensors. Thus, in contrast to direct learning from one-dimensional time series, PRISM-Block facilitates a more efficient approach to representation learning, as depicted in Figure 5.

To evaluate the computational efficiency and deployment feasibility of the proposed framework, we analysed the model’s complexity and inference speed. As shown in Table 3, the AET-FRAP model maintains an exceptionally lightweight architecture, comprising only 12,014 trainable parameters. This high efficiency is primarily attributed to the use of depthwise separable convolutions within the InceptionNeXt module, which significantly reduces parameter redundancy compared to standard convolutions. On an NVIDIA RTX 2080 Ti GPU, the model achieves an inference latency of 0.81 milliseconds per sample, fully meeting the real-time monitoring requirements for underground engineering.

### 2.5. Multivariate Prediction Methods

**Figure 6:**
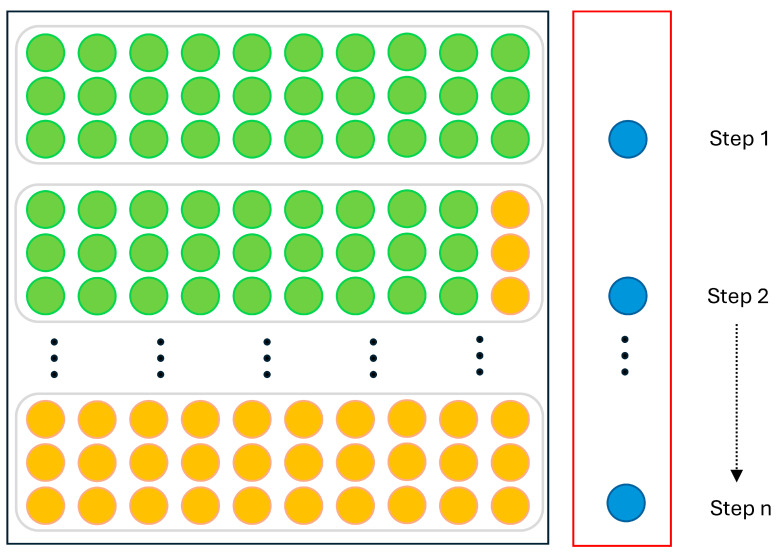
Implementation of multivariate prediction method.

AET-FRAP is a multi-input sequence-prediction model that forecasts the evolution of new variables using existing multivariate observation sequences. Figure 6 illustrates the implementation process of the prediction model. In computational Step 1, the model’s input consists of a real multivariate sequence with a defined time step length (e.g., three variables), producing a new predicted variable for the current time step as output. In computational Step 2, the input consists of single-step predictions for various variables, while the output yields the predicted values for new variables. In computational Step *n*, all inputs are the predicted multivariate values, and the output represents the prediction for the new variable at the current time step.

### 2.6. Determination of Early-Warning Thresholds

Prior studies have demonstrated that rock fractures display traits of abrupt failure. Acoustic emission signals demonstrate notable abrupt variations preceding rock fracture. This study utilizes cosine similarity to identify abrupt signals in continuous data streams, thereby establishing early-warning thresholds. Acoustic emission signals are normalized between 0 and 1, and the normalized dataset is divided into time windows of 30-step increments. As indicated in [46], the window size has a limited effect on the warning lead time. Each segment $\left(Xi\right)$ is transposed to create the vector representation ${\left(Xi\right)}^{T}$. The cosine similarity (CS) between adjacent segments ${\left(Xi\right)}^{T}$ and ${\left(Xi+1\right)}^{T}$ is determined as shown in Equation (14). The early-warning threshold for CS is established through the analysis of rock fracture test results presented in Section 4.3.

(14) $$\mathrm{CS}=\frac{\sum_{j=1}^{m}\left(Xi_{j}\cdot X\left(i+j\right)\right)}{\sqrt{\sum_{j=1}^{m}{\left(Xi_{j}\right)}^{2}\cdot \sqrt{\sum_{j=1}^{m}{\left(X{\left(i+1\right)}_{j}\right)}^{2}}}}$$

## 3. Test Setup and Data

### 3.1. Preparation of Specimens and Test Equipment

Uniaxial compression tests were performed on pillar specimens extracted from Coal Seams 3^#^, 6^#^, 8^#^, and 9^#^ at Shanxi Lingshi Xin Yi Zhi Fu Coal Industry, Lingshi Country, Shanxi, China. The coal and rock specimens were processed following the national standard, GB/T 23561-2009 [47], which outlines methods for determining the physical and mechanical properties of coal and rock. Additionally, reference was made to the ‘Recommended Methods for Rock Mechanics Testing’, developed by the International Society for Rock Mechanics’ Laboratory and Field Testing Standardization Committee. Pillars from Coal Seams 3^#^, 6^#^, 8^#^, and 9^#^ of Zhi Fu Coal Industry were collected on site. The testing equipment included a YBZS-200 automatic rock coring machine, a DQ-4 automatic rock cutter, a SHM-200 double-face grinding machine, and vernier calipers with a minimum graduation of 0.02 mm.

**Figure 7:**
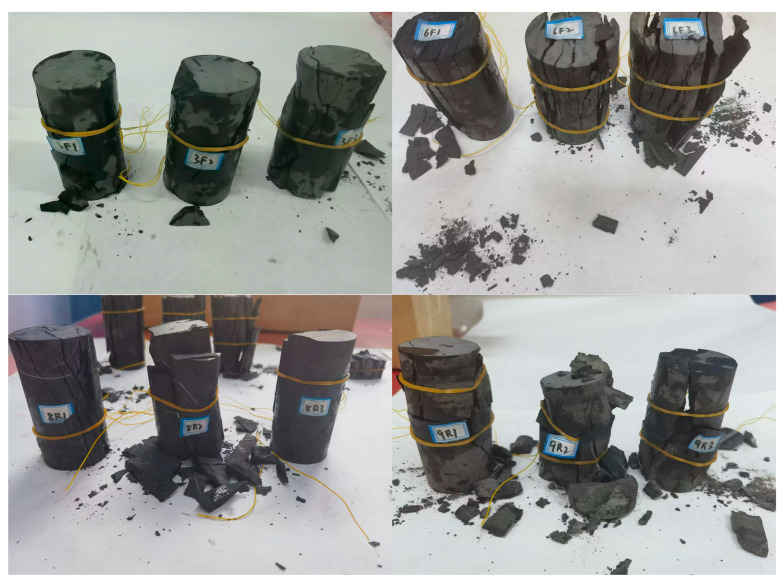
Specimens after uniaxial loading test.

**Figure 8:**
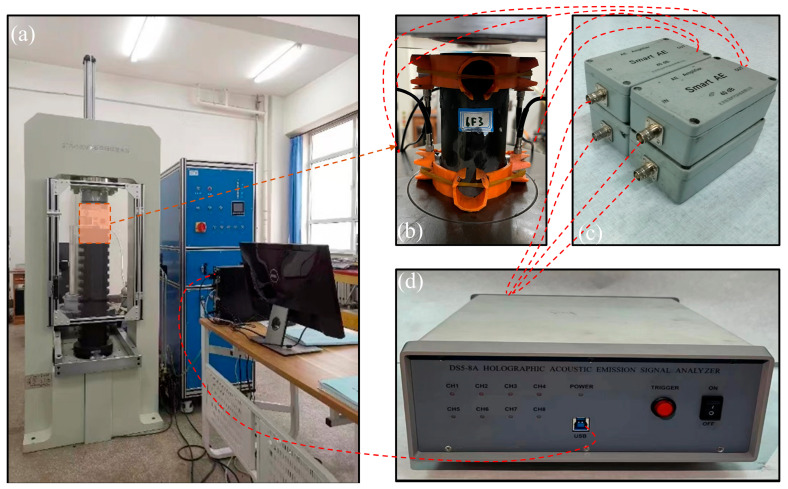
Test equipment. (**a**) ZTR-1000 Electro-Hydraulic Servo Universal Testing Machine; (**b**) specimen; (**c**) 40 dB gain module; (**d**) acoustic emission acquisition device.

Sixteen uniaxial compression tests were performed using force-controlled loading at a rate of 0.3 kN/s until macroscopic failure was observed, as shown in Figure 7. Specimen interfacing and calibration with the acoustic emission (AE) system were conducted prior to testing. Six acoustic emission sensors were strategically placed on the left, right, and rear sides of the specimen. Vaseline was utilized between the sensors and the specimen surface, secured with elastic bands to maintain stable contact. The acoustic emission acquisition system utilized was DS5-8A (Figure 8, Beijing Softland Times Scientific & Technology Co., Ltd., Beijing, China), which has a sampling frequency of 3 MHz and a preamplifier gain of 40 dB. The acoustic emission sensors exhibited a frequency response range of 100–400 kHz. Uniaxial loading and acoustic emission acquisition were synchronized to ensure consistent recording of mechanical responses and emission events. Figure 8 illustrates the uniaxial loading test system.

### 3.2. Construction of the Dataset

**Figure 9:**
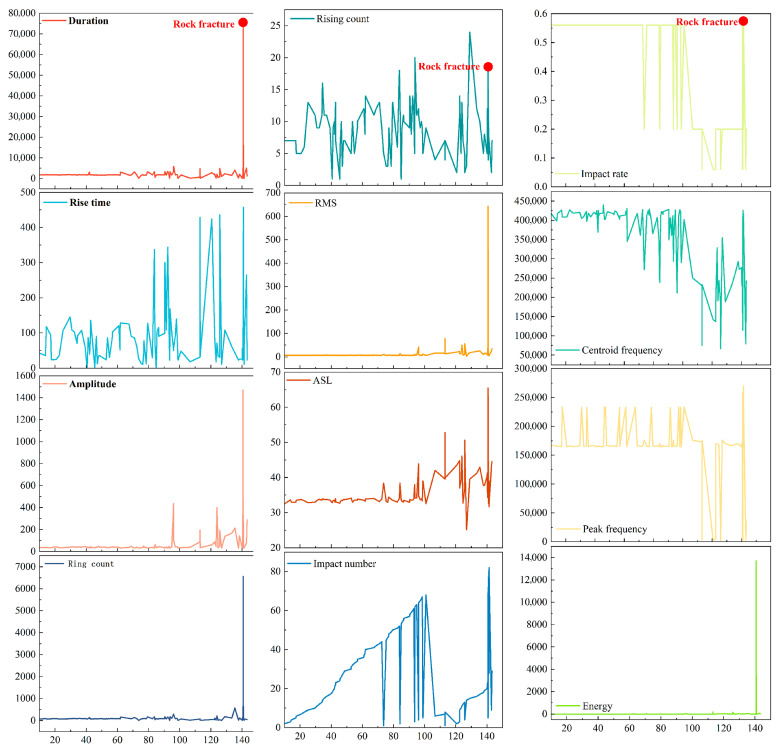
Acoustic emission characteristic parameters.

**Figure 10:**
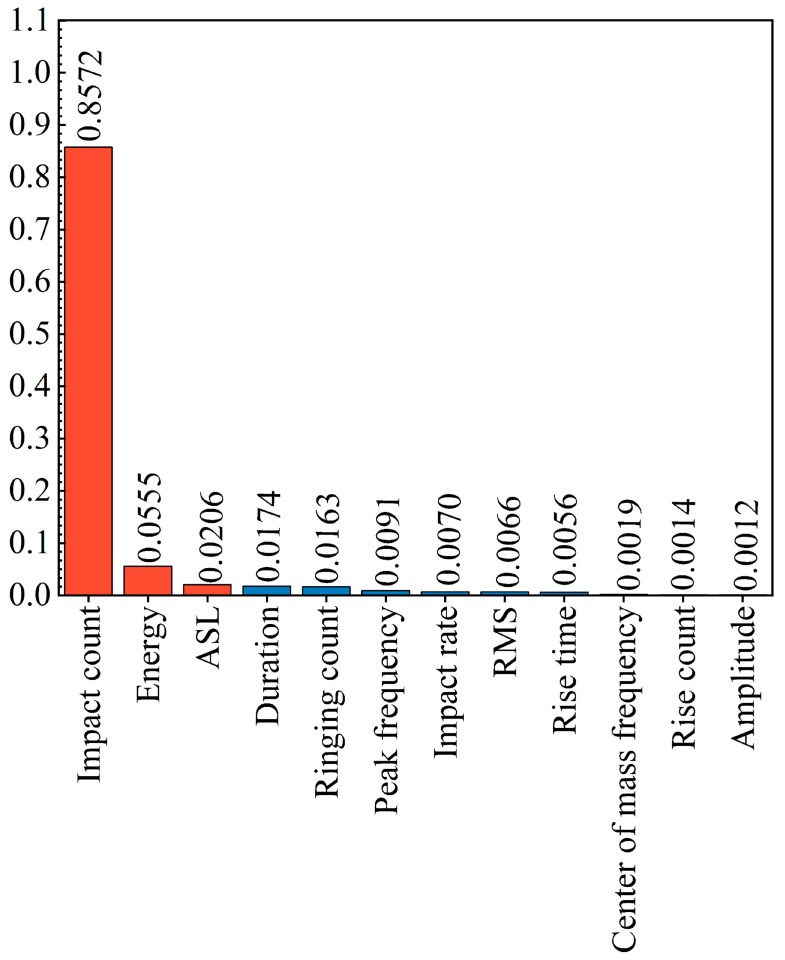
Feature importance analysis of AE parameters based on Random Forest algorithm.

The twelve parameters frequently utilized in acoustic emission (AE) studies are the following: amplitude, duration, rise time, ringing count, rise count, energy, RMS, ASL, impact count, impact rate, center of mass frequency, and peak frequency. Using the specimen from the 3F3 pillar of Coal Seam 3^#^ at Zhi Fu Coal Industry as a case study, Figure 9 illustrates the evolution of these parameters during loading. To quantitatively assess the contribution of each parameter to damage prediction and to validate the rationality of feature selection, this study employed a Random Forest algorithm for feature importance analysis (as shown in Figure 10). The results revealed a distinct hierarchy of feature contributions: impact count, energy, and ASL emerged as the most significant features, collectively accounting for over 93% of the feature importance scores. This result confirms that these parameters carry the primary trend information regarding damage accumulation.

However, despite its high statistical ranking, energy was deliberately excluded from the input feature set. As the prediction target is cumulative energy, including instantaneous energy as an input could introduce excessive correlation, causing the model to favor learning simple linear accumulation relationships over capturing complex fracture precursors. Consequently, we retained impact count and ASL as the primary statistical features. For the remaining parameters, despite their relatively low statistical scores (<2%), an analysis of waveform characteristics revealed that, based on the principle of information complementarity, rise time, rise count, peak frequency, and centroid frequency exhibited more pronounced fluctuations and abrupt changes in the period proximate to and during fracture events. Such pronounced non-stationary fluctuations often carry critical high-frequency transient information associated with crack instability, facilitating neural network capture of pre-fracture precursors [48]. Therefore, this study ultimately selected six parameters: impact count, ASL, rise time, rise count, peak frequency, and center of mass frequency as input features. This combination balances high-contribution statistical metrics with high-sensitivity waveform descriptors, ensuring both predictive accuracy and responsiveness to mutations. To mitigate bias from scale discrepancies during training, uniform standardization was applied to all input channels in subsequent model training stages.

This study designates cumulative energy as the output variable for the prediction model. Cumulative energy effectively mitigates transient noise while indicating damage accumulation and the advancement of hazard levels, demonstrating clear physical interpretability. The growth rate and inflection points of fracture initiation demonstrate significant sensitivity to critical instability states, making these effective indicators for early-warning systems. To clearly illustrate the features employed by the model and their physical magnitudes, Table 4 summarizes the units and experimental data-based ranges for all input and output variables within the AET-FRAP framework.

### 3.3. Test Environment and Parameter Settings

The computational platform running the Windows 10 operating system was employed as the testing environment. This platform featured an AMD Ryzen 5 3600 CPU, dual-channel DDR4 3200 MHz memory (16 GB × 2), and a GeForce RTX 2080 Ti GPU with 22 GB of dedicated graphics memory. Python was utilized as the programming language, with PyTorch 1.13 serving as the deep learning framework.

To eliminate potential extreme biases arising from vast scale differences between various acoustic emission parameters, all input and output channels underwent min–max normalization prior to model training. This uniformly scaled the data to the [0, 1] range, ensuring balanced gradients during backpropagation. Furthermore, to address potential multicollinearity issues in multi-parameter inputs, the proposed AET-FRAP framework effectively mitigates the sensitivity of traditional regression models to such problems by incorporating InceptionNeXt modules and a 2D periodic reshaping mechanism. This leverages the deep convolutional network’s nonlinear feature extraction capabilities, thereby ensuring robust feature learning.

The model employs the Adam optimizer for mini-batch gradient descent training, with an initial learning rate of 0.0001 and a total of 100 training epochs. Learning rate scheduling employs ReduceLROnPlateau, based on validation loss. When validation loss fails to improve over several epochs, the learning rate is reduced by a factor of 0.1. The patience value is set to 25% of the total epochs. Concurrently, an early stopping strategy is enabled: training is terminated prematurely if validation loss shows no improvement over consecutive epochs. The default batch size is 128. The experimental environment configuration is detailed in Table 5.

## 4. Results

### 4.1. Periodic Analysis of Acoustic Emission Characteristic Parameters

Empirical Mode Decomposition (EMD) is an adaptive method for decomposing nonlinear and non-stationary signals. The fundamental principle entails the construction of upper and lower envelopes at local extrema via iterative ‘sifting,’ followed by the computation of their means to progressively decompose the original sequence into multiple intrinsic mode functions (IMFs) and a slowly varying residual. Qualified intrinsic mode functions (IMFs) meet the specified criteria: approximately equal counts of poles and zero crossings, local symmetry, and near-zero envelope means. Consequently, they can be classified as narrowband AM–FM oscillatory components, characterized by well-defined instantaneous frequencies and gradually varying amplitudes. Extensive theoretical and empirical research indicates that EMD demonstrates ‘quasi-filter bank’ behavior for broadband inputs; the resulting IMFs provide a bandpass decomposition in the frequency domain, with center frequencies exhibiting an approximate bimodal distribution. This establishes a solid foundation for conducting spectral analyses on each IMF. In contrast to Fourier or wavelet transforms that depend on predefined dictionaries, EMD does not require prior basis functions. It directly generates primitives that align with the signal’s local characteristics from the data themselves, facilitating the adaptive separation of the trend, slow evolution, and multiscale oscillatory components. To ensure the convergence and physical meaningfulness of the decomposition, explicit stopping criteria were applied during the sifting process. The maximum number of sifting iterations was set to 10 to prevent over-sifting, preserving the signal’s physical amplitude variations. The sifting process for each IMF was terminated when the standard deviation (SD) between two consecutive sifting results fell below a threshold of 0.1.

Applying Fast Fourier Transform (FFT) to each intrinsic mode function (IMF) produces distinct and sharp dominant frequency peaks. Normalizing the amplitude spectra of each IMF to a common frequency axis facilitates the enhancement of cross-scale consistent periodic components while mitigating energy leakage from the trend components and transient noise. This approach effectively uncovers underlying periodic structures within complex noise and non-stationary conditions, rendering it appropriate for periodic analysis and parameter selection in acoustic emission engineering time series.

We performed regular analyses of the rise time, rise count, ASL, impact count, impact rate, center-of-mass frequency, and peak frequency for pillar 3F3 at Zhi Fu Coal Industry 3^#^. Then, we performed linear interpolation with the nonuniform sequences to derive an equidistant sampling dataset. EMD was subsequently applied to the acoustic emission characteristic parameters, decomposing the original sequence into several intrinsic modal functions (IMFs) and residuals. EMD adaptively extracts narrowband, near-zero-mean intrinsic oscillations from the data, allowing each IMF to distinctly represent the features at a specific time scale. This establishes a foundation for the independent examination of periodicity at each scale. A Fast Fourier Transform is subsequently applied to each IMF.

**Figure 11:**
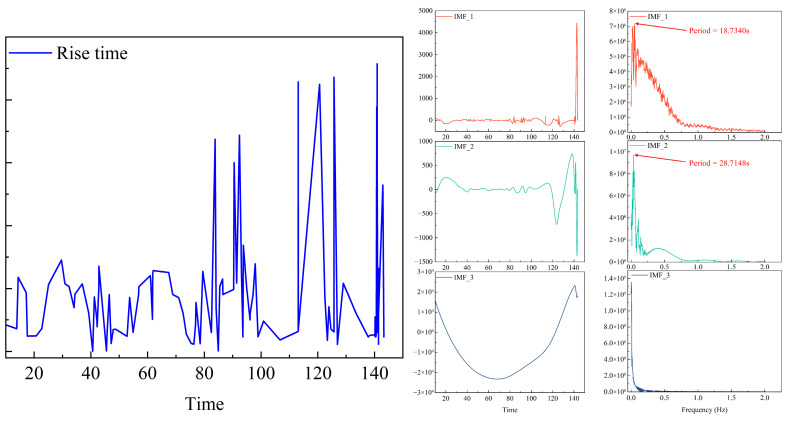
Periodic Analysis of Rise Time.

The dominant frequency is accurately determined using peak maximization and parabolic interpolation techniques. Periodic behavior is assessed based on two criteria: first, a dominant frequency threshold—if the dominant frequency is below 0.01 Hz (indicating a period greater than 100 s), then significant periodicity is not recognized; second, a relative duration constraint—if the estimated period surpasses half the recording duration, then the specimen is considered inadequately supported for that period and is excluded from analysis. The estimated principal frequency and associated period are presented for sequences that fulfill the specified criteria. The periods derived from each IMF are utilized to identify single-period or multiperiod structures. Figure 11 displays the results of the periodicity analysis for the rising-time sequence. Calculations derived from Equations (1) and (2) indicate that this sequence demonstrates significant multiperiodic characteristics, with primary periods of 18.7340 s and 28.7148 s. The IMF3 component exhibits a dominant frequency below 0.01 Hz, indicating an absence of discernible periodicity.

**Figure 12:**
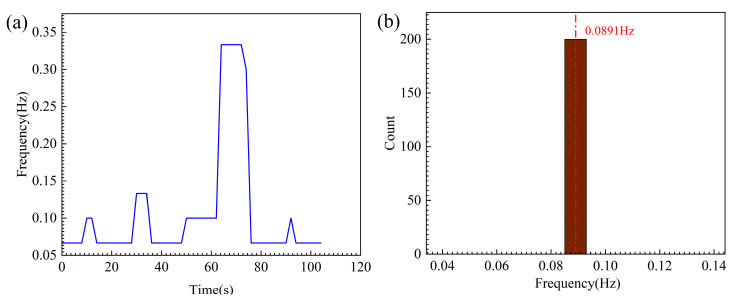
Reliability verification of periodic features. (**a**) Temporal evolution of the dominant frequency. (**b**) Frequency uncertainty analysis based on Monte Carlo simulation.

To further quantify the reliability of the EMD-FFT algorithm when processing non-stationary signals, we conducted uncertainty and repeatability analyses on the dominant feature impact count of the model. Firstly, considering the noise in the monitoring environment, we employed Monte Carlo simulations for frequency uncertainty analysis. Gaussian white noise with a signal-to-noise ratio of 20 dB was introduced into the original sequence, and the analysis was repeated 200 times. The results (as shown in Figure 12b) demonstrate that the dominant extracted frequencies consistently coincided at 0.089 Hz across all trials, exhibiting exceptional stability and confirming the algorithm’s robust immunity to measurement noise. Secondly, to rule out the possibility of random artifacts arising from short-window FFT, we employed a sliding window method to analyze the temporal evolution of the dominant frequency. As depicted in Figure 12a, the principal frequency evolution exhibits distinct ‘phased stability’ characteristics: stable low-frequency fluctuations during the initial loading phase (0–60 s), followed by a stepwise increase as failure approached (60–80 s). This continuous trajectory, consistent with the physical process of fracture initiation, confirms the excellent repeatability of the extracted periodic features, authentically reflecting the dynamic process of accumulated rock damage.

### 4.2. Analysis of Early-Warning Indicator

This study identifies cumulative energy as the primary metric for early fracture warning, employing stress as a control to assess its efficacy. Cumulative energy effectively mitigates transient noise and represents the cumulative damage process. The transition of internal cracks in specimens from initiation to instability is observed as a sudden increase and acceleration in the slope of the cumulative energy curve over time.

This physical interpretation corresponds with the phased evolution of rock failure under uniaxial compression, ensuring the clear interpretability of the metric.

**Figure 13:**
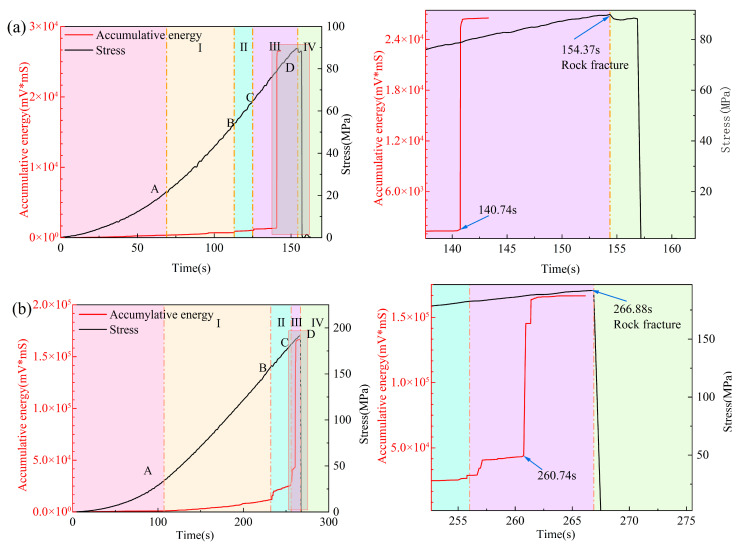
Early-warning indicator analysis: (**a**) 3F3; (**b**) 9R1.

Figure 13 demonstrates that, in the initial loading phase, cumulative energy increases progressively over time, reflecting the stage of elastic and sparse microcrack activity. The material then enters a stable propagation phase characterized by a continuously rising cumulative energy slope, with notable inflection points at moments A, B, and C. This suggests increased crack density and interaction intensity. Before final failure, cumulative energy shows a sudden increase, aligning with the stress curve nearing its peak and stabilizing. The enlarged view offers quantifiable advance times: in 3F3, the initial significant anomaly is observed at 140.92 s, with macroscopic fracture occurring at 144.72 s, resulting in an advance time of approximately 3.80 s. In 9R1, the respective moments are 261.62 s and 267.12 s, yielding a lead time of approximately 5.50 s. Both datasets exhibit a common feature, where the stress peak occurs subsequent to the cumulative energy jump prior to the final fracture of the specimen, indicating the repeatability and robustness of this indicator across various specimens. Cumulative energy is identified as a significant early-warning indicator.

### 4.3. Model Performance Evaluation

**Figure 14:**
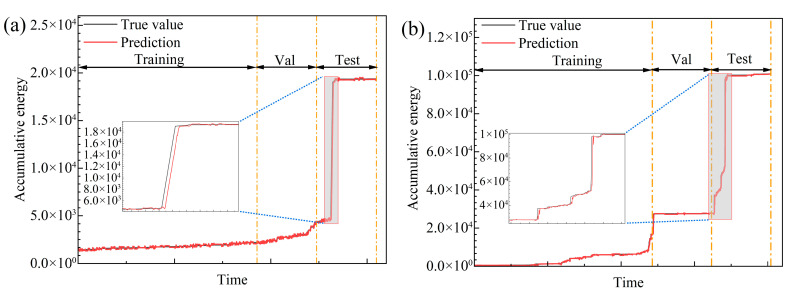
Comparison of cumulative energy prediction values and actual values. (**a**) 3F3 (**b**) 6F2.

The time series prediction model presented in Section 2 is utilized to train the early-warning indicators, using the complete experiments on 3F3, 6F2, 8R1, and 9R3 as a comprehensive example. The dataset was divided into training, validation, and test sets in a ratio of 60%:20%:20%. Standardized preprocessing and a sliding window sampling strategy were utilized during the training phase. Using specimen 3F3 and 6F2 as an example (refer to Figure 14), the model’s output curve was found to closely align with the variations in the true curve, effectively representing the key patterns of the rising segment, the peak, and the falling segment. At most time intervals, the two curves largely overlap, with only minor deviations observed during abrupt spikes and rapid inflection points. This results in a minor underestimation of peak amplitude or small phase lags near the peak.

These errors are associated with the non-stationary characteristics of the signal and the restricted visibility of information within the window, which are typical boundary conditions for time series models. The pre-peak ascent trend is effectively identified, and the post-peak decline is promptly tracked, demonstrating the model’s responsiveness and resilience to abrupt changes.

Common metrics for evaluating deep learning model performance include Mean Squared Error (MSE), Root Mean Squared Error (RMSE), Mean Absolute Error (MAE), Mean Absolute Percentage Error (MAPE), and Coefficient of Determination (R^2^). For ease of understanding, the ranges and interpretation criteria for these metrics are defined as follows: R^2^ ranges from (−∞, 1], with values closer to 1 indicating better model fit; the range for MSE, RMSE, MAE, and MAPE is [0, +∞), where values closer to 0 signify smaller prediction errors and superior model performance, as shown in Table 6. The specific calculation formulas for these metrics are as follows:

(15) $$\mathrm{MSE}=\frac{1}{n}\sum_{i=1}^{n}{\left(y_{i}-{\hat{y}}_{i}\right)}^{2}$$

(16) $$\mathrm{RMSE}=\sqrt{\mathrm{MSE}}=\sqrt{\frac{1}{n}\sum_{i=1}^{n}{\left(y_{i}-{\hat{y}}_{i}\right)}^{2}}$$

(17) $$\mathrm{MAE}=\frac{1}{n}\sum_{i=1}^{n}\left|y_{i}-{\hat{y}}_{i}\right|$$

(18) $$\mathrm{MAPE}=\frac{100\%}{n}\sum_{i=1}^{n}\left|\frac{y_{i}-{\hat{y}}_{i}}{y_{i}}\right|$$

(19) $$R^{2}=1-\frac{\sum_{i=1}^{n}{\left(y_{i}-{\hat{y}}_{i}\right)}^{2}}{\sum_{i=1}^{n}{\left(y_{i}-\bar{y}\right)}^{2}}$$

(20) $$\bar{y}=\frac{1}{n}\sum_{i=1}^{n}y_{i}$$

where *n* denotes the sample size, $y_{i}$ represents the *i*-th actual value, and ${\hat{y}}_{i}$ denotes the *i*-th predicted value.

**Figure 15:**
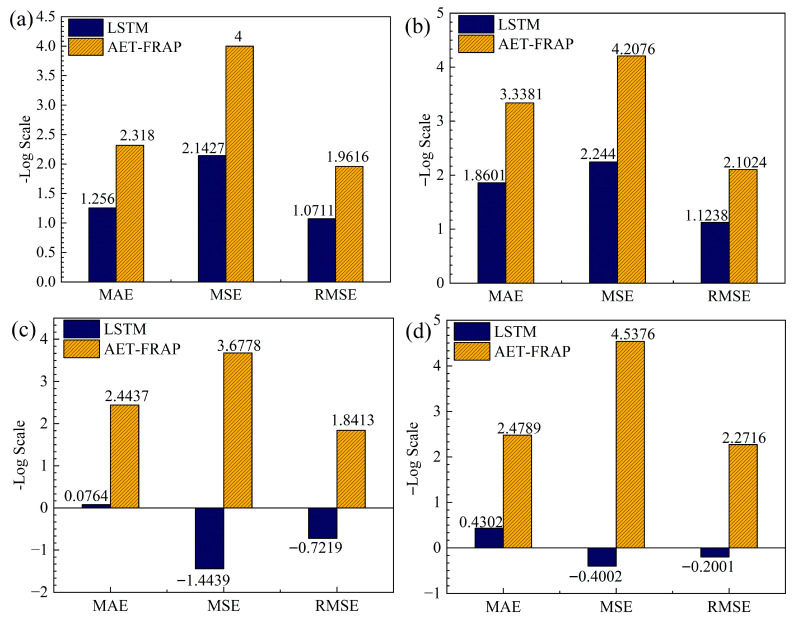
Comparison of error metrics. (**a**) Comparison results for 3F3. (**b**) Comparison results for 6F2. (**c**) Comparison results for 8R1. (**d**) Comparison results for 9R3.

Due to the notable disparities between LSTM and AET-FRAP regarding MSE, RMSE, and MAE, we illustrate these metrics on a negative logarithmic scale in Figure 15, with higher values signifying superior model performance. The figure illustrates that the −lg(MSE) of AET-FRAP is consistently distributed within the range [3.6778, 4.5376], whereas LSTM’s distribution has a range of [−1.4439, 2.244], resulting in an average difference of approximately 3.7 logarithmic units in MSE. The −lg(RMSE) distribution for AET-FRAP spans [1.8413, 2.2716], while LSTM’s is contained within [−0.7219, 1.1238], with an average gap of about 1.86 logarithmic units. Additionally, the −lg10(MAE) distribution for AET-FRAP has a range of [2.318, 3.3381], in contrast to LSTM’s range of [0.0764, 1.8601], yielding an average difference of approximately 1.86 logarithmic units.

**Figure 16:**
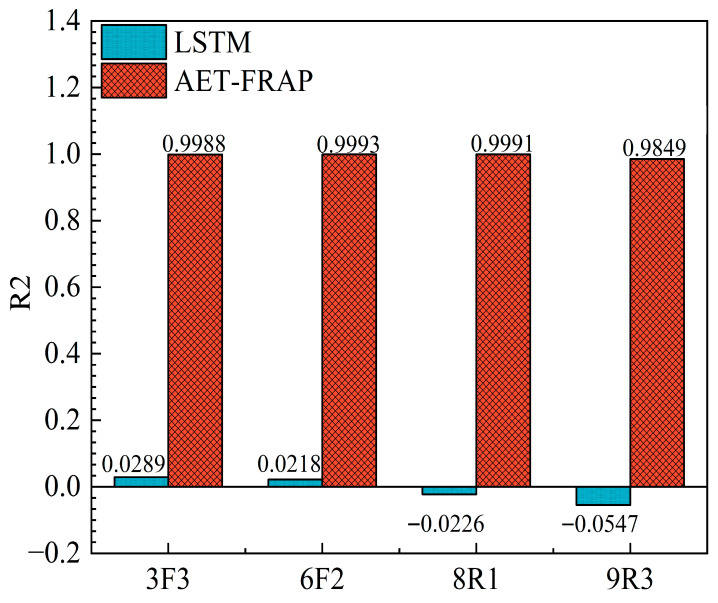
Comparison of predicted goodness-of-fit (R^2^) for different specimens.

In conjunction with the R^2^ histogram presented in Figure 16, AET-FRAP demonstrates a goodness-of-fit approaching 1 for all four specimens. In contrast, LSTM’s R^2^ hovers around 0.02 and is negative for specimens 8R1 and 9R3, signifying its insufficient explanatory capacity for overarching trends. Further analysis revealed the reasons for LSTM’s poor performance. The rock-fracture process involves extreme non-stationarity, with data distribution undergoing a drastic shift from stable phases to sudden fracture events. LSTM models, which rely on recursive historical states, are constrained by their inherent memory-retention mechanism. They tend to predict smooth trajectories based on predominantly stable historical data, rendering them incapable of accommodating the abrupt exponential surges at fracture points. This lag causes the model to entirely miss critical high-energy peaks. Mathematically, when prediction errors at these large peaks exceed the data’s inherent variance, R^2^ becomes negative, indicating the model’s failure to capture abrupt trend shifts. In contrast, AET-FRAP circumvents this recursive limitation by capturing two-dimensional temporal patterns, effectively identifying the characteristics of the abrupt process.

To further validate the superiority of the proposed framework, we contrast our findings with existing literature on rock fracture and rock burst prediction. Previous studies have extensively explored deep learning applications in this domain. Liu et al. [49] developed a CNN–LSTM-based method for the temporal prediction of rock burst hazard levels, achieving effective classification of risk evolution. Similarly, Tian et al. [50] employed deep neural networks to forecast rock burst intensity levels. However, these studies primarily focused on the discrete classification of hazard levels without explicitly modeling the continuous evolution of damage-related metrics. Furthermore, traditional recurrent models often struggle when handling the pronounced non-stationarity and abrupt changes characteristic of pre-fracture acoustic emission signals. In contrast, the proposed AET-FRAP framework achieves high-precision sequential prediction of cumulative energy and effectively captures abrupt behavioral shifts through periodic reshaping. Compared to existing classification-based approaches, this offers a more interpretable and refined early-warning scheme.

### 4.4. Early Warning for Rock Fracturing

To rigorously determine early-warning thresholds, we implemented a quantitative optimization process based on the statistical performance of precursor indicators. Within the context of rock fracture early warning, balancing two conflicting objectives is paramount: minimizing ‘false alarms’ (i.e., issuing alerts when the rock is safe) while avoiding ‘missed alarms’ (i.e., failing to issue alerts prior to fracture). To scientifically evaluate threshold efficacy, we introduced the F1-score as a composite performance metric. The F1-score represents the harmonic mean of precision and recall, calculated as follows:

(21) $$\mathrm{Precision}=\frac{\mathrm{TP}}{\mathrm{TP}+\mathrm{FP}}$$

(22) $$\mathrm{Recall}=\frac{\mathrm{TP}}{\mathrm{TP}+\mathrm{FN}}$$

(23) $$F1=2\frac{\mathrm{Precision}\times \mathrm{Recall}}{\mathrm{Precision}+\mathrm{Recall}}$$

Among these, TP (true positive) denotes the correctly identified precursors to rupture, FP (false positive) represents the stable phases erroneously identified as precursors (false alarms), and FN (false negative) signifies the missed rupture events. A higher F1-score indicates greater stability for this threshold combination.

**Figure 17:**
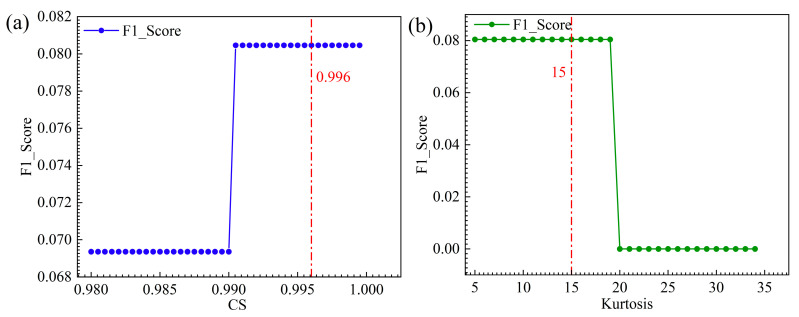
Determination of optimal collaborative early-warning thresholds based on F1-score sensitivity analysis. (**a**) F1-score sensitivity to Cosine Similarity (CS); (**b**) F1-score sensitivity to Kurtosis.

Based on this, we formulated threshold determination as an optimization problem. We defined the 5 s time window preceding the macro-fracture as the ground truth anomaly and performed a grid search within the cosine similarity (CS), with a threshold range of [0.980, 0.999] and a kurtosis threshold range of [5, 35]. To address potential minute temporal discrepancies between waveform distortion and energy release, we applied a 10-step rolling window for feature alignment. As illustrated in Figure 17 (using 3F3 as an example), the F1-score sensitivity curve reveals an optimal plateau region for detection performance. The combination of CS = 0.996 and kurtosis = 15 falls precisely within this region. Notably, this specific threshold combination achieved 100% precision on the representative sample 3F3 while maintaining an extremely low false alarm rate across other test samples. This indicates that the thresholds effectively filter environmental noise by sacrificing some detection of early, weak signals. This strategy is crucial in engineering practice as it ensures that issued alarms possess exceptionally high credibility. To rigorously evaluate the universality of the selected collaborative thresholds, we extended this early-warning criterion to all test samples. Detailed statistical results are presented in Appendix B.

Using 6F1 and 8R1 as case studies, cumulative energy was predicted via the AET-FRAP model, followed by the computation of cosine similarity (CS) and kurtosis for early-warning indicators to detect mutation points. CS quantifies the correlation of cumulative energy waveforms over time, with values approaching 1 indicating the enhanced stability of the early-warning indicator. Kurtosis measures the sharpness and impulsiveness of signal distribution, where elevated values denote an increase in short-duration, high-amplitude transient events. During loading, as the specimen progresses from the elastic stage to the damage evolution stage, crack initiation occurs, resulting in a decrease in cosine similarity and a marked increase in kurtosis. After conducting several trials, the CS threshold was established at 0.996 and the kurtosis threshold was established at 15, with a consistent value observed across 10 windows, suggesting an impending fracture event. The simultaneous selection of CS and kurtosis as collaborative early-warning indicators effectively captures both decreasing correlation and increasing sharpness precursors, thereby reducing false alarms caused by noise or sporadic events that may arise from reliance on a single indicator.

**Figure 18:**
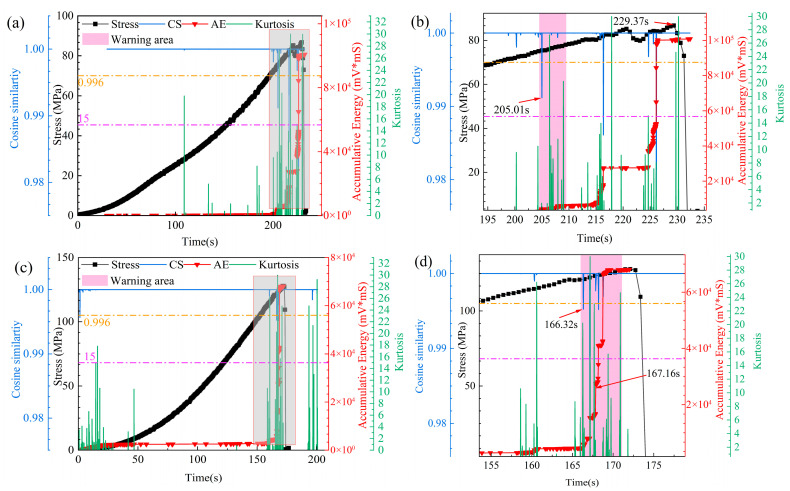
Precursor identification of fracture triggered by coordinated thresholding of cosine similarity and kurtosis: (**a**) 6F1; (**b**) local feature map of 6F1; (**c**) 8R1; (**d**) local feature map of 8R1.

Figure 18 illustrates that the overall specimen and its localized enlargement demonstrate a sustained monotonic increase in the stress curve before reaching the peak, with minor fluctuations observed. This phenomenon is generally linked to the rapid propagation and convergence of internal microcracks. As the instability phase neared, the early-warning indicators demonstrated notable and consistent synergistic alterations. The CS curve exhibited stability at a high level near 1.0 during the initial- and mid-loading stages, suggesting a relatively smooth energy release process within the rock, characterized by a strong temporal correlation. Upon entering the pink warning window, the CS value exhibited a significant stepwise decline, consistently remaining below the 0.996 threshold.

This phenomenon indicates a change in the internal damage state of the rock, transitioning from the independent nucleation of microcracks to a phase characterized by the rapid convergence and interconnection of macrocracks. During this stage, acoustic emission events exhibit greater spatial and temporal dispersion and disorder, resulting in a loss of the original stability and autocorrelation of the predicted cumulative energy sequence. This results in a significant decrease in the CS indicator.

The kurtosis curve shows a significant positive correlation during the specified purple window period. The kurtosis values remained low during the majority of the loading duration, suggesting a uniform distribution of energy release event intensities. Upon entering the early-warning window, the kurtosis values consistently surpassed the threshold of 15, resulting in a series of closely clustered high-value peaks. This corresponds directly to a fundamental change in the mechanism of energy release within the rock. The increase in kurtosis indicates the presence of multiple high-energy acoustic emission events occurring over a brief period, which is indicative of crack instability propagation and the sudden release of localized stress concentrations.

This warning strategy’s primary advantage is its ability to facilitate collaborative alerting. Individual indicators are vulnerable to disruption by random occurrences. An isolated strong acoustic emission event may temporarily cause the kurtosis to exceed established thresholds. Nonetheless, if the damage has not reached a critical threshold, the CS value will remain elevated, thus mitigating the risk of false alarms. In contrast, a gradual decrease in the CS value without a corresponding release of concentrated energy (low peak values) may indicate variations in loading conditions rather than an indication of imminent fracture. A sustained decline in CS, accompanied by a persistent increase in peak values, must remain stable across more than ten windows for the system to classify it as a high-confidence precursor to fracture. This design markedly improves the robustness and reliability of the early-warning system.

The identified mutation points inherently reflect the forward-looking nature of the AET-FRAP model, as these indicators are derived from the model’s projected values for future cumulative energy. The early-warning trigger times shown in Figure 15 (e.g., 205.01 s for specimen 6F1 and 351.81 s for specimen 8R1) occur prior to both the actual fracture times (229.37 s for 6F1 and 167.16 s for 8R1) and the moments when these precursors were clearly evident in the measured data. The realized warning lead time is the sum of the physical precursor duration and the model-predicted time, thus providing a more valuable time window for implementing intervention measures.

### 4.5. Model Generalisation Capability and Sensitivity to Noise

A model’s generalization capability and robustness against interference serve as key indicators for assessing its applicability in practical engineering scenarios. To validate the universality of the AET-FRAP framework across varying coal seam conditions and its stability in complex environments, this study conducted rigorous cross-seam generalization testing and noise sensitivity analyses. First, in an independent validation experiment, the model was trained solely using data from Coal Seam 3 (specimen 3F3) and was then directly tested on Coal Seam 8 (specimen 8R1), the data for which had not been used in the training phase. Despite differences in the physical–mechanical properties and the depositional environments between the specimens from these distinct coal seams, the model achieved an R^2^ value as high as 0.9987 on the 8R1 test set. This outcome demonstrates that the model successfully captures the underlying, universal temporal evolution patterns in the accumulation of acoustic emission energy prior to coal–rock fracture, rather than being confined to the specific characteristics of a single specimen. Consequently, it exhibits robust transferability and generalization capabilities across diverse coal seam conditions.

**Figure 19:**
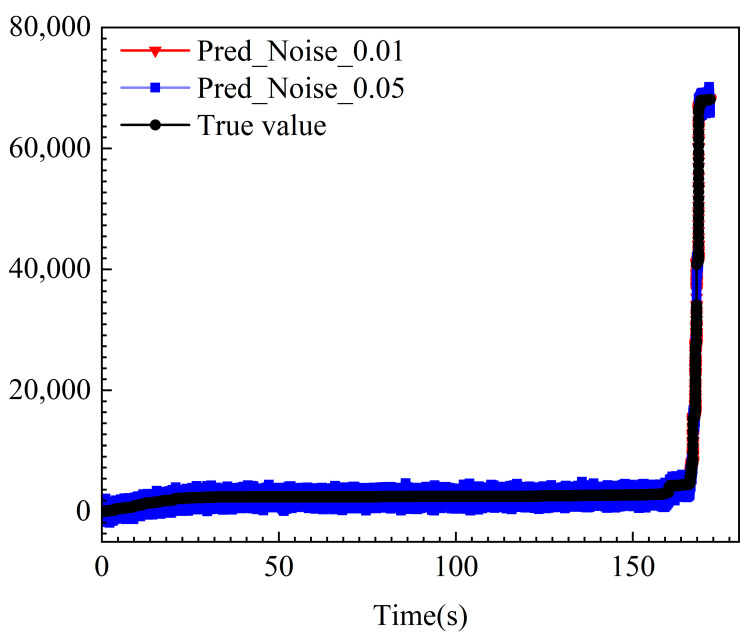
Analysis of model prediction robustness under different levels of noise interference.

Furthermore, given that the monitoring signals in actual underground engineering environments inevitably suffer from interference from ambient noise and measurement errors, assessing the model’s sensitivity to noise is paramount. We introduced Gaussian white noise at intensities of 1% and 5% of the signal amplitude into the test data to simulate varying degrees of signal contamination. The comparison of differently colored curves in Figure 19 clearly demonstrates that, even with noise superimposed, the predicted curves closely track the trends of actual values. Quantitative analysis reveals that, with 1% noise introduced, the model’s R^2^ remains at an exceptionally high level of 0.9977; even under the stronger interference of 5% noise, R^2^ still maintains a value of 0.9738. As noise intensity increases, the model’s performance exhibits only a slight and reasonable decline, consistently maintaining high accuracy above 0.97. These findings confirm that the AET-FRAP framework not only possesses outstanding generalization capabilities but also demonstrates exceptional robustness to data noise, thereby meeting the reliability requirements for early warning of rock mass fracturing in practical engineering settings. To further validate the robustness of the AET-FRAP framework across diverse geological materials and complex stress environments, cross-lithology validation was conducted using sandstone specimens under triaxial cyclic loading (see Appendix A).

## 5. Discussion

### 5.1. Periodicity of Acoustic Emission Parameters

In the present study, we aimed to examine the existence of temporal patterns in acoustic emission (AE) signals that could be leveraged for predictive applications. We selected a range of parameters—including rising time, rising count, and ASL—that demonstrate the notable fluctuations and abrupt changes that occur during the pre-fracture phase. These parameters were chosen for their capacity to convey substantial damage-state information, rather than any prior assumptions regarding their periodicity. We now perform a periodicity analysis of these parameters utilizing the EMD + FFT method.

Table 7 illustrates that periodicity is not a universal trait among all acoustic emission parameters. Specimens from Coal Seams 3^#^, 6^#^, and 9^#^ display abundant periodic structures across several parameters, indicating that their fracture processes may be primarily influenced by periodic stress accumulation and release events. Nonetheless, significant phenomena arose in specimens obtained from Coal Seam 8^#^, where nearly all metrics had no observable periodicity. The existence or nonexistence of periodicity is closely connected to the physical characteristics of the rock samples. Moreover, measures including impact rate and the number of impacts exhibited non-periodic behavior in most cases.

### 5.2. Prospects for Correlating Periodicity with the Physical Characteristics of Rock Fracturing

The intermittent occurrence of acoustic emission signals is not solely a mathematical attribute at the signal processing level; it is inferred to be intimately connected to the physical mechanisms that regulate the progression of internal damage in rock. This study primarily aims to build a prediction framework, but investigating the physical meaning of these periodicities offers valuable guidance for future research.

We hypothesize that the stable periodicity observed in the signals may correspond to certain cyclic micro-mechanical behaviors; however, verifying this theoretical link will require further investigation. The cumulative effect of these mechanical characteristics ultimately manifests macroscopically as periodic fluctuations in the acoustic emission signals. Conversely, the absence of periodicity, as commonly observed in the specimen from Coal Seam 8^#^, may indicate a more direct and catastrophic failure pathway. This may suggest that the failure mode for such rock samples is not one of gradual damage accumulation; rather, it is a rapid, brittle through-fracture. The energy is released explosively within an extremely short timeframe, thereby preventing the formation of stable periodic signals. This difference in failure mode may be rooted in intrinsic physical variations between rock samples from different coal seams, such as mineral composition and the degree of cementation.

Establishing a quantifiable correlation between the periodicity of acoustic emission signals and certain physical damage mechanisms is a formidable yet essential research avenue. The results reported here establish a basis for this undertaking. Our upcoming study will concentrate on utilizing advanced monitoring tools to correlate the periodic patterns detected at the signal level with the physical mechanisms that drive the evolution of rock microstructures. This will allow us to elucidate the physical consequences of these early-warning signs, improving the mechanistic clarity and dependability of early-warning models.

### 5.3. Research Limitations

Despite the encouraging results, this study retains certain limitations. Firstly, while uniaxial compression tests effectively simulate the primary vertical loading of mine pillars, a scale effect gap persists between laboratory specimens and in situ engineered pillars. The complex distribution of natural fractures within large-scale rock masses may introduce signal variability that small-scale samples cannot fully capture. Future research would benefit from model validation using field monitoring data. Secondly, expanding the dataset to encompass a wider variety of rock lithologies will further enhance the model’s generalizability.

## 6. Conclusions

In this study, we present and substantiate an integrated, intelligent early-warning framework, AET-FRAP, which was designed for the early detection of rock fractures to provide timely warnings. The framework addresses this challenge by utilizing acoustic emission data from uniaxial tests, including indicator selection, time series modeling, and threshold determination. The primary contributions are as follows:

1. We identify the most informative feature combinations for model inputs, focusing on the fluctuating and abrupt characteristics of various acoustic emission parameters during the pre-fracture stage. Cumulative energy, which holds clear physical significance, is the primary prediction objective. A combined EMD and FFT analysis method was utilized to perform a detailed periodic analysis of the selected feature sequences.
2. The fundamental mechanism of the AET-FRAP model involves ‘periodic reshaping’, which is used to convert one-dimensional acoustic emission signals into two-dimensional tensors. This facilitates the simultaneous capture of dynamic changes both intra-period variation and inter-period variation across various scales, resulting in enhanced feature extraction. The model integrates a lightweight InceptionNeXt module to maintain computational efficiency. The experimental results indicate that this approach surpasses the LSTM baseline model in prediction accuracy across various test samples, with R^2^ nearing 1.0 and notable decreases in MSE, RMSE, and MAE compared with the baseline, effectively capturing the sudden rise in cumulative energy during the pre-fracture stage.
3. This study proposes a two-stage early-warning strategy to mitigate the susceptibility of traditional single-point thresholds to noise interference. This method simultaneously computes cosine similarity (CS) and kurtosis in high-precision prediction sequences instead of directly assessing predicted values. This shifts the warning task from the sensitive detection of ‘single-point values’ to the robust recognition of ‘sequential behavioral patterns’, thereby significantly improving the reliability of precursor capture. The validation of the experimental data confirmed that the coordinated trigger conditions, i.e., CS < 0.996 and kurtosis > 15, were maintained over 10 consecutive windows. These criteria integrate physical precursors—specifically, signal instability due to damage accumulation—with quantitative metrics, thereby effectively reducing sporadic false alarms caused by random noise.

In this study, we have validated the framework’s effectiveness from a data-driven perspective and proposed a theoretical explanation for periodicity; however, its microscopic physical mechanisms warrant further investigation. Future work will focus on integrating geomechanical numerical simulations to rigorously verify the physical connection between the observed signal periodicity and internal damage evolution within rock. This approach will transcend the existing theoretical assumptions and further consolidate the physical foundations of early-warning models.

## Figures and Tables

**Table 1:** Comparative analysis of dominant periods identified using different resampling methods.

IMF	Period (Cubic Spline)	Period (Linear)	Relative Difference [%]
IMF1	5.6956	5.6956	0.00
IMF2	6.8727	6.8727	0.00
IMF3	14.0408	14.0408	0.00
IMF4	28.2963	28.2963	0.00
IMF5	35.1479	35.1479	0.00
IMF6	56.8713	56.8713	0.00

**Table 2:** Complexity of Different Convolution Types k, C, H and W denote kernel size, number of channels, height and width, respectively.

Conv. Type	Params	Flops
Conventional conv.	K^2^c^2^	2k^2^c^2^HW
Depthwise conv.	K^2^c^2^	2k^2^CHW
Inception conv.	(2k + 9) C/8	(2k + 9) CHW/4

**Table 3:** Detailed network architecture and computational complexity analysis of the AET-FRAP model. B denotes batch size. Within PRISMBlock, 1D temporal features are reshaped into 2D tensors based on identified principal periods. P represents period length (reflecting intraperiod variation), while F denotes frequency (reflecting interperiod variation). Model parallel processing corresponds to k such tensors for the top-k principal periods.

Stage	Input Shape	Output Shape	Description
Input	(B, 30, 6)	—	Batch size B, Seq len 30, Features 6
Embedding	(B, 30, 6)	(B, 30, 64)	Data + Time + Position Embedding
PRISMBlock (2D Reshape)	(B, 30, 64)	(B, 64, P, F)	Reshaped into Top-k 2D tensors based on periods
InceptionNeXt (2D Conv)	(B, 64, P, F)	(B, 64, P, F)	Depthwise Separable Convolutions
Aggregation	(B, 64, P, F)	(B, 30, 64)	Flatten and adaptive weighting
Output projection	(B, 35, 64)	(B, 5, 1)	Linear projection to target dimension
Total Parameters	—	12,014	Extremely lightweight architecture
Inference Time	—	0.81 ms/sample	Real-time capability on RTX 2080 Ti

**Table 4:** Definitions and statistical ranges of input and output variables for the AET-FRAP framework.

Variable Name	Type	Unit	Range (Min–Max)
Rise time	Input	uS	0.33–457.33
Rise count	Input	-	1–24
ASL	Input	dB	19.62–72.06
Impact count	Input	-	1–82
Center of mass frequency	Input	kHz	66.42–440.14
Peak frequency	Input	kHz	0.00–398.44
Cumulative energy	Output	mV · mS	1.81–2.90 × 10^5^

**Table 5:** Experimental environment configuration.

Environment Configuration	Version Model
Operating System	Windows 10 Professional
GPU	RTX 2080TI (22 GB)
Cpu	AMD Ryzen5 3600
Programming Language	Python 3.8
Deep Learning Framework	Pytorch 1.13
Cuda Verson	Cuda 11.6

**Table 6:** Performance comparison of LSTM and AET-FRAP.

	Model	R^2^	MSE	RMSE	MAE	MAPE
3F3	LSTM	0.0289	0.0072	0.0849	0.0554	1.9397
	AET-FRAP	0.9988	0.0001	0.0109	0.0048	3.4688
6F2	LSTM	0.0218	0.0057	0.0752	0.0138	35.6339
	AET-FRAP	0.9993	0.000062	0.0079	0.00459	3.049
8R1	LSTM	−0.0226	27.7925	5.2719	0.8386	5.2142
	AET-FRAP	0.9991	0.00021	0.0144	0.0036	3.3987
9R3	LSTM	−0.0547	2.5129	1.5852	0.3714	2.5867
	AET-FRAP	0.9980	0.000029	0.0053	0.0033	1.0266

**Table 7:** Periodic Analysis Results for Acoustic Emission Parameters.

	Duration	Rising Count	Impact Rate	Rise Time	RMS	Centroid Frequency	Amplitude	ASL	Peak Frequency	Ring Count	Impact Number	Energy
3F1	4	4	3	4	5	5	5	5	6	2	3	4
3F3	4	0	0	3	2	4	3	4	3	3	2	3
6F1	1	4	0	2	2	2	2	2	1	2	0	2
6F2	4	5	1	4	5	5	5	4	5	3	4	3
6F3	4	5	0	3	4	5	4	5	5	7	2	4
8R1	1	5	0	1	1	1	2	1	0	1	0	1
8R2	1	0	0	0	0	0	0	0	0	0	0	0
8R3	0	0	2	0	0	0	0	1	1	0	2	0
9R1	5	4	0	3	4	4	5	3	3	4	0	4
9R2	4	5	0	4	3	4	5	5	5	4	1	3
9R3	1	0	1	4	1	1	2	1	3	5	1	1

The values in the table represent the number of significant periodic components identified within the corresponding parameter’s time series.

**Figure A1:**
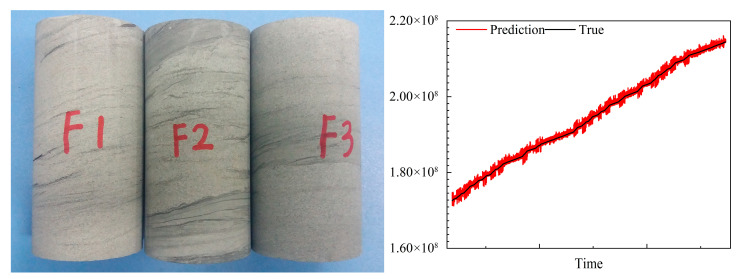
Predicted results for sandstone specimen F1 under triaxial cyclic loading.

## Data Availability

The datasets generated during and/or analyzed during the current study are available from the corresponding author on reasonable request.

## Appendix A. Cross-Lithology Generalisation Validation

To further validate the robustness of the AET-FRAP framework across diverse geological materials and complex stress environments, this study introduced fine sandstone specimens—distinct from the aforementioned coal rock samples—for cross-lithology validation. Unlike the uniaxial compression tests on coal rock, this experimental series employed a more intricate triaxial cyclic loading path: applying 4.3 MPa confining pressure followed by multi-stage axial cyclic loading and unloading until specimen failure. Despite confronting dual challenges of lithological variation and loading path complexity, the AET-FRAP model demonstrated exceptional adaptability. Taking specimen F1 as an example, the prediction results are shown in Figure A1. The red predicted curve closely aligns with the black actual cumulative energy curve, demonstrating the model’s ability to accurately fit the long-term energy growth trend while effectively capturing local fluctuations induced by cyclic loading. Quantitative evaluation reveals an R^2^ of 0.9975 across the entire test set, with a MAPE as low as 0.21%. This outcome confirms that the ‘Periodic Reshape’ mechanism proposed by AET-FRAP effectively extracts the deep temporal characteristics prevalent in rock damage processes, demonstrating universal early warning capability across rock types and loading conditions.

## Appendix B. Detailed Statistical Performance of Early-Warning Thresholds

To rigorously evaluate the universality of the selected collaborative thresholds (CS < 0.996, Kurtosis > 15), we extended this early-warning criterion to all 11 (Sample 3F2 was excluded from the analysis due to incomplete data resulting from experimental issues) test samples. Detailed statistical results are presented in Table A1. Analysis indicates that the AET-FRAP framework successfully triggered valid early warnings in the vast majority of samples (75%), maintaining exceptionally high precision in all successful cases. Only the 6F2 sample recorded a negligible number of false alarm signals. This confirms the model’s outstanding capability in filtering environmental noise and non-destructive disturbances. Although this stringent threshold strategy resulted in false negatives (TP = 0) for certain samples (e.g., 6F3, 8R1, 8R3), this aligns with the engineering safety principle established in this study: prioritising the assurance of extremely high credibility for every issued alert to prevent on-site trust crises caused by high false alarm rates.

**Table A1:** Detailed performance metrics for all evaluated samples under the collaborative threshold.

Sample ID	TP	FP	FN	Precision	Recall	F1-Score	Result Analysis
3F1	5	0	162	1.000	0.030	0.058	Valid warning
3F3	7	0	160	1.000	0.042	0.081	Valid warning
6F1	8	0	159	1.000	0.048	0.091	Valid warning
6F2	6	1	161	0.857	0.036	0.069	False alarm
6F3	0	0	167	0.000	0.000	0.000	Missed alarm
8R1	0	0	167	0.000	0.000	0.000	Missed alarm
8R2	7	0	160	1.000	0.042	0.081	Valid warning
8R3	0	0	0	0.000	0.000	0.000	Missed alarm
9R1	6	0	161	1.000	0.036	0.069	Valid warning
9R2	9	0	158	1.000	0.054	0.102	Valid warning
9R3	5	0	162	1.000	0.029	0.058	Valid warning
